# Single-cell spatial landscape of aggrephagy activity stratifies hepatocellular carcinoma neutrophils and delivers a 5-gene diagnostic panel for patient stratification

**DOI:** 10.1016/j.tranon.2026.102893

**Published:** 2026-07-04

**Authors:** Wanju Jiang, Kai Wang, Guoshu Li, Qiqi Zhang

**Affiliations:** a Department of General Surgery, Jinshan Hospital, Fudan University, Shanghai, 201508, China; b Department of Pulmonary Rehabilitation, Shanghai Yangzhi Rehabilitation Hospital (Shanghai Sunshine Rehabilitation Center), School of Medicine, Tongji University, Shanghai, China; c Department of Hepatopancreatobiliary Surgery, Shanghai East Hospital, School of Medicine, Tongji University, Shanghai 200120, China

**Keywords:** Aggrephagy, Tumor-associated neutrophils, Hepatocellular carcinoma, Tumor microenvironment, Immunotherapy

## Abstract

**Background:**

Hepatocellular carcinoma (LIHC) features a complex tumor microenvironment (TME) where tumor-associated neutrophils (TANs) show significant plasticity. The role of aggrephagy—selective autophagy of protein aggregates—in shaping neutrophil heterogeneity and LIHC progression remains poorly understood.

**Methods:**

We integrated scRNA-seq (183,671 cells), spatial transcriptomics, and bulk datasets (TCGA, GSE39791). Neutrophils (n=12,547) were re-clustered into six subsets, and aggrephagy activity was quantified via UCell scores. Analysis included pseudotime trajectories, cell–cell communication, metabolic scoring, and machine-learning-based feature selection, followed by in vitro functional validation.

**Results:**

Aggrephagy activity was significantly elevated in tumor tissues compared with adjacent normal tissues (P < 0.001) and showed strong cell-type specificity, with TANs among the most enriched populations. High-aggrephagy neutrophils exhibited an undifferentiated state, preferential tumor enrichment, and a positive correlation with transcriptomic risk scores. Trajectory analysis positioned these cells at an early differentiation branch and revealed dominant neutrophil-to-stroma signaling through the CCL3–CCR1, SPP1–CD44, and ANXA1–FPR1 axes. Metabolically, high-aggrephagy neutrophils displayed enhanced inflammatory and epithelial mesenchymal-transition programs alongside suppressed oxidative phosphorylation. Integrative network analysis identified a five-gene diagnostic panel (SQSTM1, WDFY3, DOCK4, CD177, LIMK2) with robust performance across bulk cohorts (AUC 0.83–0.91). Among these, LIMK2 marked a highly interactive neutrophil subset and functionally promoted tumor cell proliferation, survival, migration, and invasion in vitro.

**Conclusion:**

Aggrephagy is associated with a pro-tumorigenic, metabolically reprogrammed neutrophil state in LIHC. The LIMK2-centered gene panel provides a robust framework for subset identification and nominates candidate targets for future autophagy- and neutrophil-directed studies.

## Highlights

• Single-cell and spatial profiling mapped aggrephagy activity in LIHC.
• High-aggrephagy neutrophils mark immature, tumor-enriched TAN states.
• HAS neutrophils displayed enhanced inflammatory and epithelial mesenchymal-transition programs alongside suppressed oxidative phosphorylation.
• Machine learning defined a 5-gene panel for LIHC patient stratification.
• LIMK2 knockdown restrained HCC proliferation, migration and invasion in vitro.

## Graphical abstract

**Figure:**
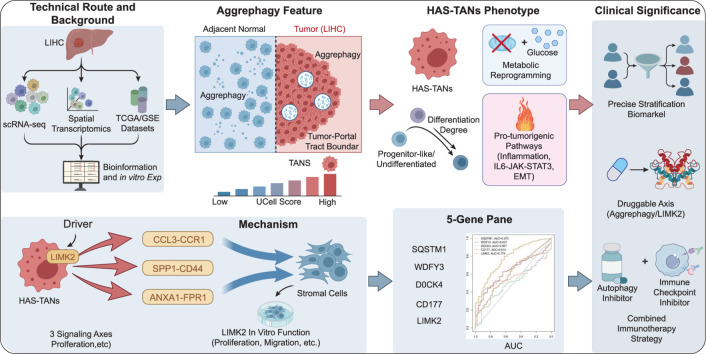

## Introduction

Hepatocellular carcinoma (LIHC) is one of the leading causes of cancer-related deaths worldwide [[1], [2], [3]]. Its highly heterogeneous tumor microenvironment (TME) significantly impacts disease progression, treatment response, and prognosis [[4], [5], [6]]. As the most abundant myeloid immune cells in the TME, neutrophils have traditionally been regarded as a terminally differentiated homogeneous population, primarily responsible for nonspecific defense functions [[7], [8], [9], [10]]. However, recent single-cell transcriptomic evidence has revealed that tumor-associated neutrophils (TANs) exhibit substantial heterogeneity in phenotype, function, and developmental trajectory, encompassing multiple polarized states such as pro-tumorigenic (N2) and anti-tumorigenic (N1) [[11], [12], [13]]. They are deeply involved in the malignant progression of LIHC through mechanisms including cytokine secretion, extracellular matrix remodeling, and immune suppression mediation [14,15]. Despite the widespread attention on neutrophil heterogeneity, the systematic characterization of their subset composition, molecular features, and functional regulatory networks in LIHC remains lacking, which limits the precise implementation of individualized immunotherapeutic strategies [[16], [17], [18]].

Aggrephagy, a crucial branch of selective autophagy, recognizes and clears ubiquitinated protein aggregates via autophagy receptors, thereby maintaining intracellular protein homeostasis [19,20]. Recent studies have indicated that abnormal autophagic flux not only drives metabolic reprogramming and survival of tumor cells but also reshapes the functional states of immune cells, affecting their secretory profiles, survival, and cytotoxic capabilities [[21], [22], [23]]. However, at the single-cell level, several key questions remain unaddressed: how aggrephagy activity is associated with neutrophil heterogeneity, whether it can serve as an informative molecular marker for subset classification, and whether it exhibits spatial distribution preferences in the LIHC microenvironment [[24], [25], [26]]. Given the close cross-regulation between autophagy and inflammatory signals, analyzing the expression profiles of aggrephagy-related genes (AGGREPHAGY-ATGs) in TANs is expected to uncover novel functional subsets and the molecular basis underlying their dual pro-carcinogenic/anti-carcinogenic roles [27,28].

With the integrated application of high-throughput single-cell RNA sequencing (scRNA-seq) and spatial transcriptomics, researchers can simultaneously capture transcriptional states and spatial location information at single-cell resolution, providing unprecedented opportunities to elucidate immune cell heterogeneity and functional molecular markers [29,30]. Based on large-scale LIHC single-cell atlases, this study integrates multi-algorithm autophagic activity scoring, pseudotime trajectory reconstruction, and cell communication inference to systematically identify neutrophil subsets highly associated with aggrephagy and validate their spatial distribution characteristics and clinical significance [[31], [32], [33]]. We hypothesize that aggrephagy activity may represent an informative molecular dimension for classifying functional subsets of TANs [34,35]. The high-autophagy subset may exhibit an undifferentiated, pro-inflammatory-fibrotic phenotype and associate with adverse clinical features [36].

In summary, this study aims to construct a single-cell autophagy atlas of LIHC neutrophils, identify and validate reproducible and translatable aggrephagy-related marker combinations, thereby laying a theoretical foundation for the subsequent development of precise immunotherapeutic interventions based on neutrophil reprogramming or autophagy regulation.

## Materials and methods

### Acquisition and preprocessing of transcriptomic data

RNA-seq raw counts matrices and corresponding clinical information of the TCGA-LIHC (The Cancer Genome Atlas-Liver Hepatocellular Carcinoma) project were downloaded from the UCSC Xena public data portal (https://xenabrowser.net/), including 374 tumor tissue samples (T) and 50 paired adjacent normal tissue samples (N). To ensure cross-platform comparability, the counts data were first uniformly converted to Transcripts Per Million (TPM) values using the StringTie-TPM pipeline, followed by log₂(x+1) transformation to reduce heteroscedasticity. Similarly, an external validation dataset GSE39791 (platform: Affymetrix HG-U133A Plus 2.0), containing 72 liver cancer samples and 72 paired non-tumor tissue samples, was obtained from the NCBI-GEO database (https://www.ncbi.nlm.nih.gov/geo/). Background correction, normalization, and probe annotation of the raw microarray CEL files were performed in R software (v4.1.3) using the RMA algorithm. Finally, a log2-transformed expression matrix was generated for subsequent differential and survival analyses.

### Collection and quality control of single-cell data

Single-cell RNA sequencing data were derived from three GEO series: GSE136103 (11 normal liver tissue samples), GSE290925 (12 liver cancer tissue samples), and GSE166635 (2 liver cancer tissue samples), all using the 10 × Genomics Chromium platform. All raw FASTQ files were aligned and quantified against the GRCh38-2020-A genome using CellRanger v6.1.2 to generate cell and gene counts matrices. Subsequent quality control and integration were uniformly performed in R 4.1.3 using Seurat 4.3.0 with the following filtering criteria: mitochondrial gene proportion < 20%, hemoglobin gene proportion < 3%, unique molecular identifier (UMI) count between 200–20,000, and detected gene count between 200–6,000. Normalization was conducted using the LogNormalize method with a scaling factor of 10,000. Highly variable genes were selected using the FindVariableFeatures function to identify the top 2,000 highly variable genes. Confounder regression was performed via the ScaleData function, with cell cycle scores (S.Score, G2M.Score) and mitochondrial proportion as covariates to reduce non-biological variation. Batch effect correction was implemented using the Harmony v0.1.0 algorithm, with dataset source as the batch variable and the first 30 principal components (PCs) for integration. Dimensionality reduction and clustering were carried out by first running RunPCA (dims = 1:30), then visualizing with RunUMAP (min.dist = 0.3, spread = 1.0), and finally calling FindClusters with the Louvain algorithm at a resolution of 0.8, resulting in 32 initial cell clusters. Differential gene identification was based on the Wilcoxon rank-sum test, with parameters set as minimum detection proportion min.pct = 0.1, log2 fold-change threshold of 0.25, and significance level P < 0.05. Additionally, spatial transcriptomic sections (Visium platform) were obtained from the GEO project GSE203612 (4 liver cancer tissue samples) for subsequent spatial localization validation of autophagy scores.

To explicitly control cross-dataset technical variation, batch correction was performed before downstream clustering and visualization using Harmony on the shared PCA space, with dataset source specified as the batch variable. Integration quality was assessed by examining whether the post-correction embeddings were driven predominantly by biological lineage markers rather than dataset identity and whether major canonical markers remained restricted to their expected cell populations after correction. A formal parallel reconstruction using Seurat anchor-based integration was not adopted as the primary analytical workflow in the current study; accordingly, all reported clustering results should be interpreted as Harmony-based estimates.

### Definition of the aggrephagy gene set

The aggrephagy-related gene set was downloaded from the Molecular Signatures Database (MSigDB v2025.1), with the specific entry GOBP_AGGREPHAGY (https://www.gsea-msigdb.org/gsea/msigdb/human/geneset/GOBP_AGGREPHAGY.html), containing 35 human genes. This curated gene set, compiled through literature review, includes components of the ubiquitin-proteasome system, selective autophagy receptors (e.g., SQSTM1/p62, NBR1, OPTN), and lysosome-related proteins, which were used for subsequent functional scoring and heterogeneity analysis.

### Cell type annotation

After obtaining 183,671 high-quality cells, manual annotation was performed based on well-recognized classic markers: Hepatocytes: EPCAM, KRT18, KRT19, ALB;Fibroblasts: DCN, THY1, COL1A1, COL1A2;Endothelial cells: PECAM1, CLDN5, FLT1, RAMP2;T/NK cells: CD3D/E/G, TRAC; NKG7, GNLY, NCAM1, KLRD1;B cells: CD79A, IGHM, IGHG3, IGHA2 (plasma cells additionally highly express JCHAIN);Myeloid cells: LYZ, MARCO, CD68, FCGR3A;Mast cells: KIT, MS4A2, GATA2;Neutrophils: FCGR3B, CSF3R. Annotation results were validated through multi-dimensional visualization, including UMAP dimensionality reduction plots, bubble heatmaps, and violin plots, to ensure consistency between marker expression of each lineage and clustering structure.

### Neutrophil subset refinement

For refined analysis of neutrophil subsets, 12,547 cells annotated as neutrophils were first extracted, followed by re-execution of an independent analysis pipeline: sequentially performing NormalizeData, FindVariableFeatures (selecting 1,500 features), ScaleData, RunPCA (dims = 1:20), RunUMAP (dims = 1:20, min.dist = 0.2), and FindClusters (resolution = 0.6). Ultimately, 6 subsets (N0–N5) were obtained, which were used for subsequent heterogeneity and functional state evaluation.

### Pseudotime trajectory analysis

Monocle 2 (v2.24.1) was used to infer the differentiation trajectory of neutrophil subsets. Key parameters included: dimensionality reduction method = DDRTree, max_components = 2, norm_method = "log", and expression_family = "negbinomial". The high aggrephagy expression group (HAS) was set as the starting point, and differentiation potential was calculated using CytoTRACE to evaluate the dynamic relationship between autophagic activity and differentiation maturity.

### Cell communication inference

The CellChat v1.5.0 tool was used to systematically evaluate the intercellular communication network. First, the Seurat-normalized expression matrix was used as input to construct an object via the createCellChat function. Subsequently, the identifyOverExpressedGenes and identifyOverExpressedInteractions functions were executed to screen for significant ligand-receptor pairs. When setting computeCommunProb, the minimum number of cells expressing ligand-receptor complexes was set to ≥ 10, and interactions with a communication probability ≥ 0.05 were retained using filterCommunication. At the pathway level, computeCommunProbPathway and aggregateNet were called to generate circle plots, heatmaps, and bubble plots, which respectively display the overall network, incoming/outgoing signal intensities, and differential communication patterns between HAS and LAS neutrophils.

### Aggrephagy activity scoring

To reduce algorithm dependency, four independent strategies were combined to quantify aggrephagy activity at the single-cell level: 1. AUCell (v1.20.0): Area Under the Curve method with auc.thresh = 0.05;2. UCell (v2.0.0): Rank-based statistics with n.features set to 35;3. Seurat AddModuleScore: Control gene set size = 100 with 100 random samplings;4. singscore (v1.14.0): "Total" scoring mode.

After z-score normalization of the four scores, the highest consistency was observed between UCell and AUCell (Pearson r = 0.92), so UCell scores were finally selected for downstream analysis. Neutrophils were divided into high-autophagy group (HAS, top 33%), medium-autophagy group (DTAS, middle 34%), and low-autophagy group (LAS, bottom 33%) based on tertiles. The same strategy was repeated in spatial sections to validate spatial distribution characteristics.

### Human tissue specimens and ethical approval

Tumor tissues and paired adjacent non-tumorous tissues were obtained from five patients with HCC presenting with liver metastasis. Adjacent normal tissues were collected at a distance of at least 3 cm from the tumor margin to avoid tumor cell contamination. All tissue samples were acquired during surgical resection procedures performed at our institution between May 2019 and April 2025. Immediately after excision, tissue specimens were snap-frozen in liquid nitrogen and subsequently stored at −80°C to preserve RNA integrity for downstream molecular analyses. This study was approved by the Institutional Review Board (IRB) of our hospital. Written informed consent was obtained from all patients prior to tissue collection and study enrollment, in accordance with the Declaration of Helsinki.

### RNA extraction and quantitative real-time PCR (qRT–PCR)

Total RNA was extracted from cultured cells and frozen tissue specimens using TRIzol reagent (Invitrogen) according to the manufacturer’s instructions. RNA concentration and purity were assessed using a NanoDrop 2000 spectrophotometer (Thermo Fisher Scientific), and only RNA samples with an A260/A280 ratio between 1.8 and 2.0 were used for subsequent experiments. For cDNA synthesis, 1 µg of total RNA per sample was reverse-transcribed using the PrimeScript RT reagent kit (Takara Bio). Quantitative real-time PCR was performed using SYBR Premix Ex Taq (Takara Bio) on a QuantStudio™ 5 Real-Time PCR System (Applied Biosystems). The PCR cycling conditions were as follows: initial denaturation at 95°C for 30 s, followed by 40 cycles of denaturation at 95°C for 5 s and annealing/extension at 60°C for 30 s. Relative gene expression levels were calculated using the 2^−ΔΔCt method, with GAPDH serving as the internal reference gene. Each experiment was performed in triplicate, and all data represent the mean values of three independent biological replicates.

### Cell culture and authentication

All cell lines used in this study were authenticated by short tandem repeat (STR) profiling and routinely tested for mycoplasma contamination. Cells were maintained in Dulbecco’s Modified Eagle Medium (DMEM) supplemented with 10% fetal bovine serum (FBS) and 1% penicillin–streptomycin solution (all from Gibco). Cells were cultured at 37°C in a humidified incubator containing 5% CO₂. When cell confluence reached approximately 80%, total RNA was isolated for subsequent analyses. LIMK2 expression in cell lines was quantified by qRT–PCR following the same procedures described for tissue samples. All experiments, including RNA extraction and qRT–PCR assays, were independently repeated three times to ensure reproducibility.

### siRNA transfection

Small interfering RNAs (siRNAs) targeting LIMK2 and corresponding negative control siRNAs were synthesized by TsingKe Biotechnology. Each siRNA was reconstituted in nuclease-free water to a final concentration of 10 µM. Hep 3B and Hep G2 cells were seeded into six-well plates at a density of 2 × 10⁵ cells per well and incubated overnight to allow cell attachment. For transfection, siRNAs were mixed with Lipofectamine™ 3000 transfection reagent (Invitrogen) in Opti-MEM reduced-serum medium according to the manufacturer’s protocol. After incubation at room temperature for 15 min, the siRNA–lipid complexes were added to the cells. After 6 h, the transfection medium was replaced with fresh complete DMEM. Cells were harvested 48 h post-transfection for RNA extraction. Knockdown efficiency of LIMK2 was evaluated by qRT–PCR using GAPDH as an internal control. LIMK2 expression was reduced by more than 70% compared with the control group (p < 0.01, Student’s t-test). Each experiment was independently repeated three times.

### Cell proliferation assay

Cell proliferation was assessed using the Cell Counting Kit-8 (CCK-8; Dojindo). Briefly, cells were seeded into 96-well plates at a density of 3,000 cells per well and transfected with siRNAs as described above. Cell viability was measured at 24, 48, 72, and 96 h after transfection. At each time point, 10 µL of CCK-8 solution was added directly to each well, followed by incubation at 37°C for 2 h. Absorbance was measured at 450 nm using a Synergy H1 microplate reader (BioTek). Each condition was assessed with five technical replicates. Cell proliferation was expressed as fold changes relative to the baseline absorbance measured immediately after cell seeding.

### Flow cytometric analysis of apoptosis

Apoptosis was analyzed 48 h after siRNA transfection using an Annexin V-FITC/propidium iodide (PI) apoptosis detection kit, following the manufacturer’s instructions. Cells were harvested and gently washed twice with phosphate-buffered saline (PBS). Stained cells were immediately analyzed using a BD FACSVerse™ flow cytometer (BD Biosciences). Early apoptotic cells were defined as Annexin V–positive and PI–negative, while late apoptotic cells were defined as Annexin V–positive and PI–positive. Flow cytometry data were analyzed using FlowJo software.

### Cell migration and invasion assays

Cell migration and invasion assays were performed using Transwell chambers with 8 µm pore-size polycarbonate membranes (Corning). For migration assays, cells were resuspended in serum-free medium and seeded into the upper chambers at a density of 5 × 10⁴ cells per chamber. Medium containing 10% FBS was added to the lower chambers as a chemoattractant. After incubation at 37°C for 24 h, non-migrated cells on the upper surface of the membrane were gently removed with a cotton swab. Migrated cells on the lower surface were fixed with 4% paraformaldehyde and stained with 0.1% crystal violet. Cells were counted under a light microscope in five randomly selected fields per chamber. For invasion assays, the procedure was identical except that the Transwell membranes were pre-coated with Matrigel (BD Biosciences) prior to cell seeding. Incubation, fixation, staining, and quantification were performed as described for the migration assays.

### Machine-learning feature selection and anti-overfitting strategy

To minimize overfitting during feature prioritization, we adopted a consensus-based strategy rather than relying on a single classifier. Candidate genes were ranked in the training framework by seven complementary algorithms (decision tree, random forest, GBM, Boruta, ABESS, XGBoost, and LASSO), and only genes reproducibly retained across methods were advanced for downstream panel construction. This multi-algorithm intersection was combined with internal cross-validation during model training and with subsequent evaluation in an independent external bulk cohort (GSE39791), thereby reducing the likelihood that the final signature reflected model-specific instability or cohort-specific noise. No additional independent bulk cohort beyond TCGA and GSE39791 that simultaneously provided comparable LIHC tumor/non-tumor transcriptomic data and sufficient annotation for our analysis pipeline was available in the current study.

### Statistical analysis

All downstream analyses and plotting were performed in the R 4.1.3 environment. Comparison of differences between groups for continuous variables was conducted using the Wilcoxon rank-sum test or Kruskal-Wallis test; the χ² test was used for categorical variables. Correlation analysis was implemented using Pearson or Spearman methods. In survival analysis, the optimal cutoff value was first determined using the auto-cutoff function of the survminer package, followed by Kaplan–Meier survival curve plotting and log-rank test. The Cox proportional hazards model was used to calculate the hazard ratio (HR) and 95% confidence interval (CI). Multiple test correction was performed using the Benjamini-Hochberg method, with a significance threshold set at P < 0.05 (FDR < 0.05).

## Results

### Construction of single-cell atlas and annotation of nine liver microenvironment cell types

**Fig. 1:**
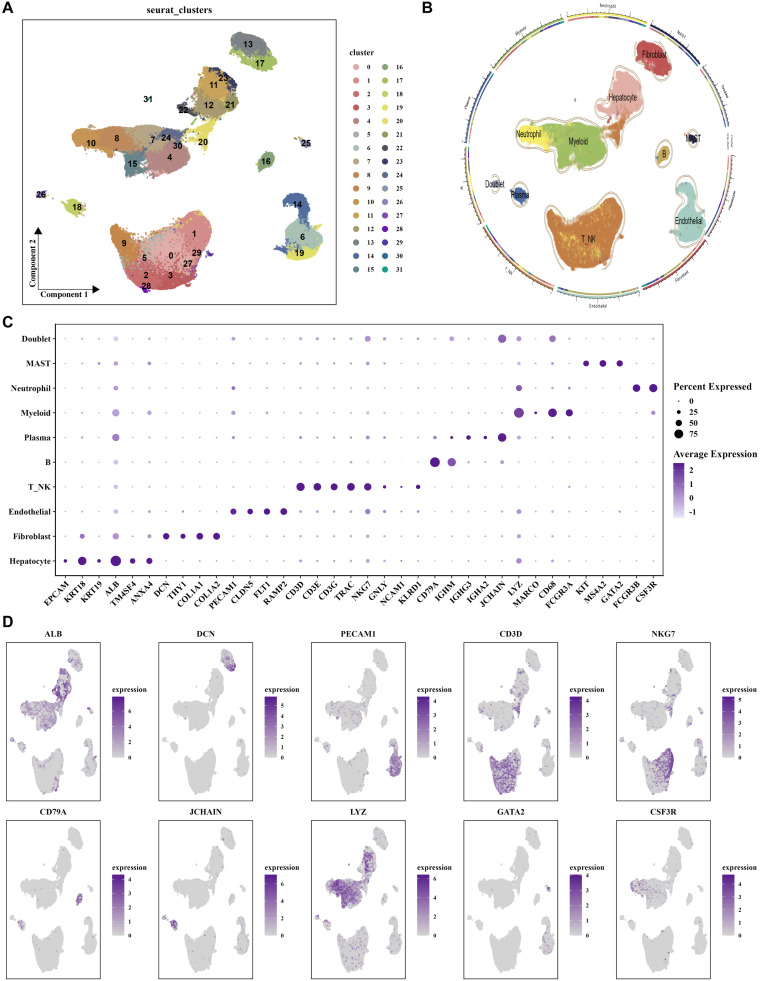
**Single-cell classification in LIHC,** (A–B) UMAP plots showing cell clusters and manual cell-type annotations. (C–D) Bubble heat-map and feature UMAPs displaying canonical marker genes across the nine major cell types.

After quality control and dimensionality reduction, a total of 183,671 cells were retained and clustered into 32 Seurat clusters (Fig. 1A). Based on classic markers, 9 major cell types were annotated: hepatocytes (ALB+), fibroblasts (DCN+), endothelial cells (PECAM1+), T/NK cells (CD3D/NKG7+), B cells (CD79A+), plasma cells (JCHAIN+), myeloid cells (LYZ+), neutrophils (CSF3R+), and mast cells (GATA2+) (Fig. 1A). UMAP visualization showed the distribution of each cluster, as well as populations such as Doublet, Plasma, and T_NK (Fig. 1B). Fig. 1C presents the average expression levels and expression percentages of markers including ANXA4, TA, and DCN across cell types such as mast cells, Doublets, and neutrophils (Fig. 1C). Subsequently, the expression distributions of markers (ALB, PECAM1, DCN, CD3D, NKG7, CD79A, LYZ, GATA2, CSF3R) on UMAP were respectively displayed (Fig. 1D).

After Harmony correction, the integrated atlas was resolved primarily according to canonical cell identity rather than obvious dataset-specific partitioning, supporting effective attenuation of major batch structure before clustering. Within the neutrophil compartment, cells from multiple scRNA-seq series contributed to the six recovered subsets and occupied overlapping manifold regions, arguing against the interpretation that these clusters were generated mainly by cohort-specific technical effects. Nevertheless, because a formal side-by-side benchmark against Seurat anchor-based integration was not performed in the present study, subtle differences in local neighborhood topology or cluster boundaries attributable to integration framework cannot be fully excluded.

### Multi-algorithm validation reveals significantly increased aggrephagy activity in tumors with cell type specificity

**Fig. 2:**
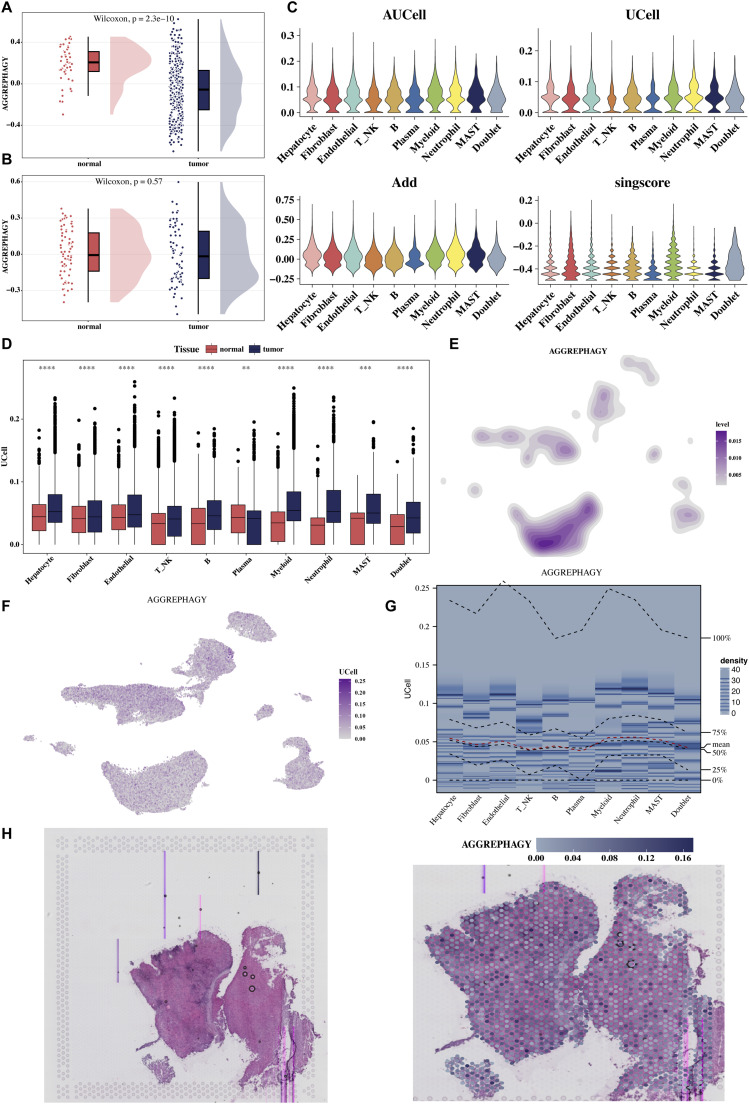
**Aggrephagy-functional analysis,** (A–B) Violin-box plots of bulk aggrephagy scores (TCGA and GSE39791) between tumor and adjacent normal tissues. (C) Violin plot comparing aggrephagy scores calculated by four independent algorithms in scRNA-seq data. (D) Box-plot of score differences between tumor and normal for each cell type. (E–G) UMAP density, standard UMAP and probability-density heat-maps of single-cell aggrephagy scores. (H) H&E image and matched aggrephagy-score overlay from spatial transcriptomics.

In the two bulk cohorts (TCGA and GSE39791), ssGSEA was used to quantify aggrephagy scores. Aggrephagy scores in TCGA tumor tissues were significantly higher than those in adjacent normal tissues (P<0.001), while GSE39791 showed an increasing trend without reaching statistical significance (Fig. 2A-B). In single-cell datasets, four algorithms (AUCell, UCell, ssGSEA, Vision) were used to calculate aggrephagy activity, showing good overall distribution and algorithm consistency (Fig. 2C). Based on UCell scores, tumor-vs-adjacent normal differences across the 9 major cell types were analyzed, with the most significant differences observed in hepatocytes and T/NK cells (Fig. 2D, P<0.01). Gradient coloring of aggrephagy scores on single-cell UMAP showed that cells with high activity were concentrated in the tumor region and exhibited cluster-specific distribution (Fig. 2E-G). Mapping UCell scores to spatial transcriptomics revealed significantly increased scores in tumor regions, with high-activity signals enriched along the portal tract-tumor boundary (Fig. 2H).

### High-aggrephagy neutrophil subsets exhibit undifferentiated phenotype, tumor enrichment, and positive correlation with TRS

**Fig. 3:**
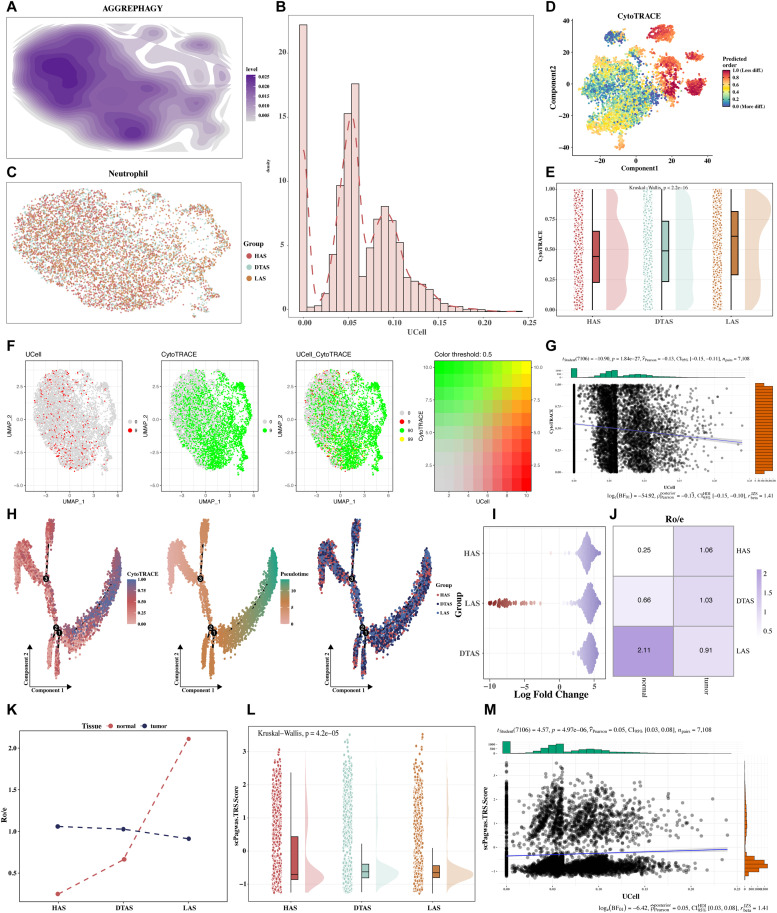
**Neutrophil-focused analysis,** (A–C) Neutrophil UMAPs colored by aggrephagy score, the three aggrephagy groups and bar plot of score distribution. (D–E) CytoTRACE differentiation scatter plot and rain-cloud plot of differentiation differences among the three groups. (F–G) Feature plots of differentiation degree vs. aggrephagy score and their correlation scatter plot. (H) Monocle2 pseudotime trajectory colored by differentiation, pseudotime and aggrephagy groups. (I) MiloR differential abundance results. (J–K) Ro/e enrichment heat-map and scatter plot. (L–M) scPagwas outputs: rain-cloud plot of TRS scores and their correlation with aggrephagy scores.

Aggrephagy (AGGREPHAGY) scores of neutrophils in tumor cells were significantly higher than those in normal cells. After isolating these neutrophils for analysis: dimensionality reduction and scoring were first performed to divide them into high (HAS), medium (DTAS), and low (LAS) aggrephagy activity groups (Fig. 3A-C); differentiation degree was then quantified using CytoTRACE, with scatter plots showing cell distribution in Component1 and Component2, and marking differentiation order (0.0-1.0) and cell counts (40, 20, etc.) (Fig. 3D); Fig. 3E shows significant differences in differentiation degrees among the three groups via Kruskal-Wallis test (p<2.2e-16), presenting the distribution of CytoTRACE values (0.00-1.00). Fig. 3F displays their UMAP distributions: aggrephagy scores (UCell, -2.5-10.0) on the left and CytoTRACE values (0.0-0.25) on the right, allowing observation of cell aggregation characteristics; correlation analysis in Fig. 3G showed a negative correlation between the two (Student (7106)=-10.90, p=1.84e-27, r=-0.13, CI95%[-0.15,-0.11], npairs=7108), which was further validated by supplementary analysis (ρ=-0.13, CI95% HDI [-0.15,-0.10]). Trajectory analysis using monocle2 was conducted, with Fig. 3H presenting the differentiation path via trajectory plots and marking the distribution and differentiation direction of cells in the three groups; MiloR and Ro/e analyses showed that the HAS group was enriched in tumor tissues while the LAS group was enriched in normal tissues, with differences presented via Ro/e values (0.5-2.0) and group comparisons, and statistical tests indicated significant effects of tissue origin (Fig. 3I-K). Finally, scPagwas analysis showed a positive correlation between aggrephagy scores and TRS scores: scatter plots display the relationship between TRS scores and Log Fold Change, and box plots compare TRS scores (0-3) among the three groups, confirming the characteristic that higher aggrephagy scores correspond to higher TRS scores (Fig. 3L-M).

Taken together, these findings support a model in which elevated aggrephagy is linked to an early neutrophil differentiation state through coordinated maintenance of proteostasis and metabolic adaptation rather than through a single downstream effector. The enrichment of aggrephagy-core genes such as SQSTM1 and WDFY3 in HAS cells suggests enhanced clearance of ubiquitinated protein cargo and damaged intracellular components, which may preserve cellular fitness during lineage transition. In parallel, the suppression of oxidative phosphorylation together with the relative enrichment of glycolytic, pentose phosphate, and arginine–proline metabolic programs indicates a shift toward a stress-adapted biosynthetic state capable of supporting inflammatory activation. Consistent with this interpretation, the concomitant upregulation of IL6–JAK–STAT3 and inflammatory-response pathways suggests that cytokine signaling is integrated with aggrephagy-associated metabolic rewiring, thereby stabilizing an immature yet tumor-adaptive neutrophil phenotype. Alternative explanations should nevertheless be considered. Because CytoTRACE and pseudotime reconstruction are inferential and derived from cross-sectional transcriptomic similarity, the HAS position may also reflect preferential retention of stress-adapted neutrophils in tumor niches, transient activation programs, or delayed terminal maturation under inflammatory and metabolic pressure, rather than a direct effect of aggrephagy in driving dedifferentiation.

Functionally, this association was reflected at multiple biological levels rather than being confined to transcriptomic stratification alone. At the cell-intrinsic level, high aggrephagy was linked to a less differentiated neutrophil state together with suppression of oxidative phosphorylation and relative enrichment of glycolytic, pentose phosphate, and arginine–proline programs, indicating a shift toward stress adaptation, biosynthetic support, and inflammatory readiness. At the intercellular level, these cells displayed reinforced outgoing communication toward endothelial cells and fibroblasts through the CCL3-CCR1, SPP1-CD44, and ANXA1-FPR1 axes, consistent with enhanced capacity to remodel stromal niches. At the tissue level, their preferential localization at the portal tract–tumor boundary and their positive association with TRS suggest that elevated aggrephagy is linked to functionally relevant consequences, including fibro-inflammatory amplification, immune-excluded microenvironmental organization, and a tumor-adaptive phenotype.

### High-autophagy neutrophils remodel metabolic and signaling networks, with the pro-inflammatory-fibrotic axis dominating

**Fig. 4:**
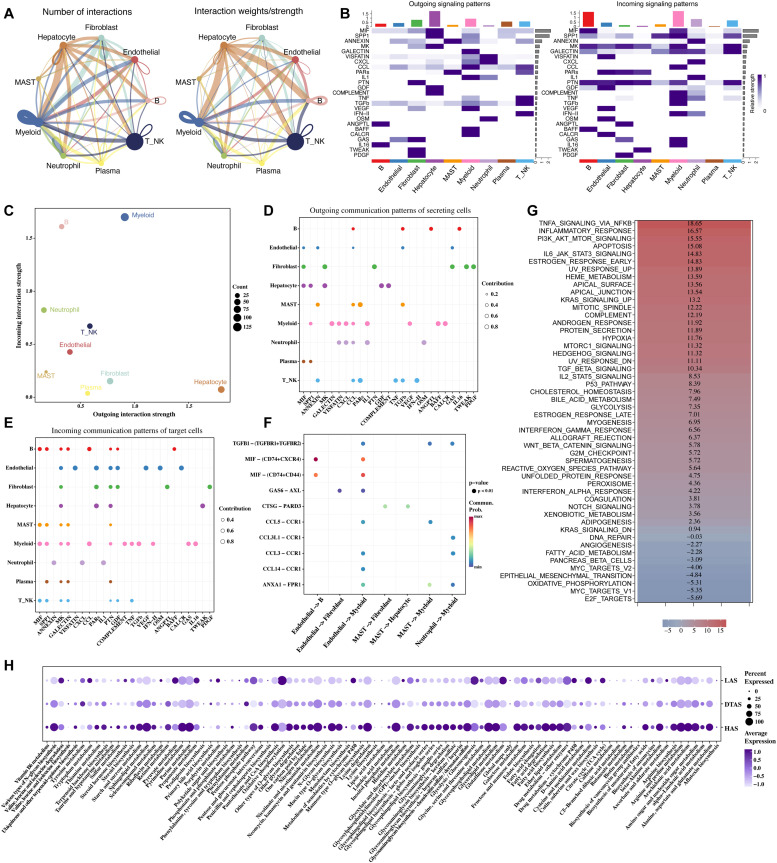
**Cell-cell communication and functional analyses,** (A–C) Circle plots of global communication (left: count-weighted; right: weight-weighted), incoming/outgoing heat-maps and scatter plots per cell type. (D–E) Bubble plots of pathway-specific incoming vs. outgoing signals for each cell type. (F) Bubble plot highlighting interactions between the three neutrophil groups and other cells. (G) Bar plot of differential Hallmark pathway activity (HAS vs. LAS). (H) Bubble plot of KEGG metabolic pathways across the three neutrophil groups.

An interaction map of 183,671 cells was constructed based on a ligand-receptor library. Endothelial cells and fibroblasts formed the core "hubs", T/NK cells and myeloid cells served as secondary signal sources, while Doublets and mast cells (MAST) were almost silent (Fig. 4A-C). Bubble plots showed that endothelial cells had high outgoing signals in both the TGFB1-(TGFBR1/2) and SPP1-CD44 axes, whereas fibroblasts exhibited the strongest incoming signals in the SPP1-(ITGAV+ITGB1) and ANXA1-FPR1 axes (Fig. 4D-E). Clusters 24, 27, and 30 were merged into "Neutro-3group" to focus on their interactions with hepatocytes, endothelial cells, and fibroblasts. Circos plots and heatmaps revealed that three axes—CCL3L1-CCR1, CSF3R-CSF3, and ANXA1-FPR1—contributed more than 70% of the weight, with the signal direction mainly from neutrophils to stromal cells (Fig. 4F). GSVA was used to calculate the scores of 50 Hallmark pathways. The high aggrephagy group (HAS) showed significant upregulation of pathways such as "Inflammatory-Response", "IL6-JAK-STAT3", and "Epithelial-Mesenchymal-Transition", while "Oxidative-Phosphorylation" and "Fatty-Acid-Metabolism" were downregulated (Fig. 4G). A total of 28 glucose-lipid-amino acid metabolism pathways were screened from KEGG. HAS hepatocytes and tumor endothelial cells ranked highest in "Glycolysis", "PPP" (Pentose Phosphate Pathway), and "Arginine-Proline" metabolism; in contrast, LAS fibroblasts and normal hepatocytes were enriched in "Fatty-Acid-Degradation" and "Oxidative-Phosphorylation". Metabolic reprogramming was positively correlated with autophagy scores, and detailed results are shown in Fig. 4H. Notably, these signaling programs are also compatible with established immunosuppressive circuits in LIHC. The CCL3/CCL3L1–CCR1 axis may amplify recruitment and positioning of suppressive myeloid and stromal elements, thereby reinforcing a cytokine-rich niche already shaped by IL6–STAT3 and TGF-β signaling. The SPP1–CD44 axis is consistent with matrix remodeling, endothelial activation, and adhesive stromal cross-talk that favor T-cell exclusion and persistence of a fibrotic tumor-supportive microenvironment. Likewise, ANXA1–FPR1 signaling may sustain neutrophil-directed inflammatory relay programs and paracrine communication with stromal compartments, which in turn can cooperate with hypoxia- and lactate-associated immune dysfunction. Together, these axes suggest that HAS neutrophils are not acting outside canonical LIHC immune evasion pathways, but are functionally embedded within broader networks of stromal activation, immune exclusion, and tumor-promoting inflammation.

### hdWGCNA identifies autophagy-coordinated module M1 and 421 core genes in neutrophils

**Fig. 5:**
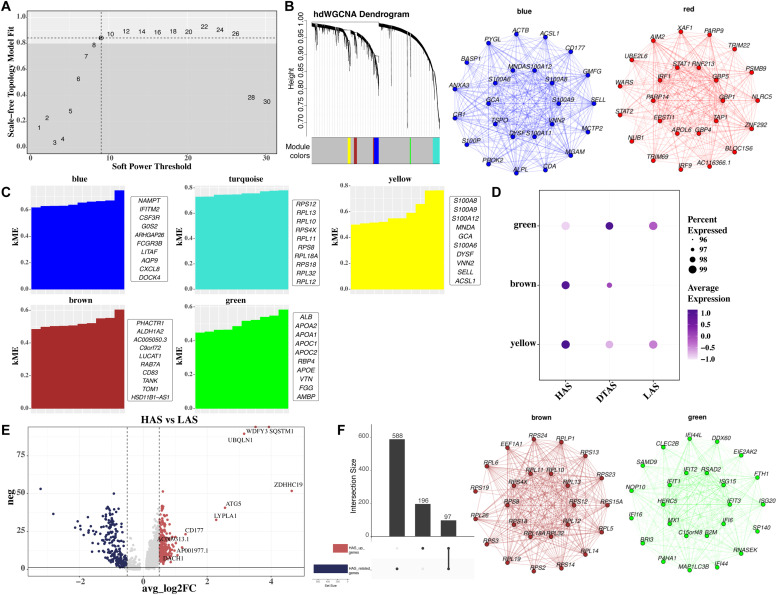
**Gene-module analysis,** (A) Scale-free topology power selection plot. (B) Hierarchical cluster tree of gene modules after dynamic cutting. (C) kME values per module. (D) Bubble heat-map of module eigengene scores across the three neutrophil groups. (E) Volcano plot of differentially expressed genes between HAS and LAS. (F) Upset plot intersecting HAS-up genes with the six co-expression modules.

hdWGCNA was used to screen for highly variable genes in three groups of neutrophils (n=9,558). When the soft-thresholding power was set to 9, the scale-free topology fitting R² was greater than 0.9, ensuring the robustness of the co-expression network (Fig. 5A). Six gene modules (M1–M6, containing 58–312 genes each) were obtained through dynamic cutting. The module eigengene distance dendrogram showed that M1 and M3 were highly similar, M4 formed an independent branch, and the hierarchical clustering was clear (Fig. 5B-C). Module eigengene (ME) values were used to quantify module activity. All six modules showed significantly higher activity in neutrophils of the HAS group than in the LAS group (Wilcoxon test, FDR<0.001), among which M1 (312 genes) had the most significant upregulation, suggesting a high coordination with aggrephagy (Fig. 5D). A total of 1,347 HAS-upregulated genes were identified with the criteria of |log2FC|>0.58 and FDR<0.05. Volcano plots showed that the top-ranked genes included core inflammatory-autophagic molecules such as S100A8/9, MMP9, and LCN2 (Fig. 5E). Upset analysis was performed to find the intersection between the 1,347 HAS-differentially expressed genes (HAS-DEGs) and the six modules, leading to the identification of 421 "core genes" shared by M1, M3, and M5. Enrichment analysis indicated that these core genes were significantly involved in "NOD-like receptor signaling", "autophagy", and "neutrophil degranulation", providing candidates for subsequent functional validation (Fig. 5F).

### Machine learning with cross-validation identifies a 6-gene signature explaining 82% of autophagy score variation

**Fig. 6:**
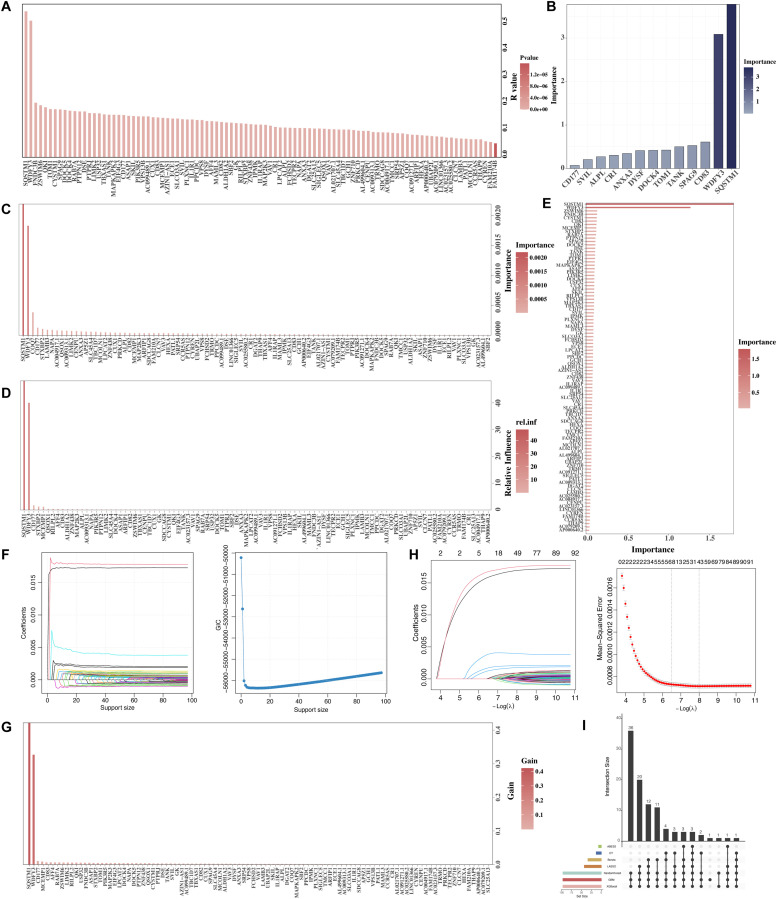
**Machine-learning gene selection,** (A) Lollipop plot showing correlation of 25 candidate genes with aggrephagy scores. (B–H) Importance rankings from seven ML algorithms (decision tree, random forest, GBM, Boruta, ABESS, XGBoost, LASSO). (I) Upset plot of genes consistently selected by all seven algorithms.

Spearman correlation tests were conducted between each of the 25 intersecting genes and the single-cell aggrephagy score (UCell). Among them, 22 genes showed significant correlations (P<0.001), and SQSTM1, WDFY3, CD177, IRAK2, and TANC2 ranked among the top with correlation coefficients >0.45 (Fig. 6A). In the training set, multiple machine learning methods—including decision tree, random forest, GBM (Gradient Boosting Machine), Boruta, ABESS (Adaptive Best Subset Selection), XGBoost, and LASSO (Least Absolute Shrinkage and Selection Operator)—were run simultaneously. SQSTM1, WDFY3, CD177, TANK, and QKI consistently ranked among the top 5 important genes (Fig. 6B-H). The important genes output by each algorithm were intersected, and the Upset plot showed that 6 key genes were shared by all 7 methods: SQSTM1, WDFY3, CD177, TANK, QKI, and IRAK2. The expression of these 6 genes could explain 82% of the variance in aggrephagy scores, providing a refined signature for subsequent prognostic model construction and mechanism validation (Fig. 6I).

### The 5-gene diagnostic panel is highly expressed in tumor tissues, and LIMK⁺ neutrophils drive pro-inflammatory-fibrotic signals

Importantly, external validation of the diagnostic genes in the current study was limited to one independent cohort beyond TCGA, namely GSE39791. We therefore interpret the observed AUC range as supported by cross-platform replication in a single external dataset rather than by multiple independent validation cohorts. To reduce overfitting, candidate selection was restricted to genes consistently prioritized across seven machine-learning algorithms, the training process incorporated cross-validation, and diagnostic performance was then re-evaluated in GSE39791 rather than estimated only in the discovery dataset.

**Fig. 7:**
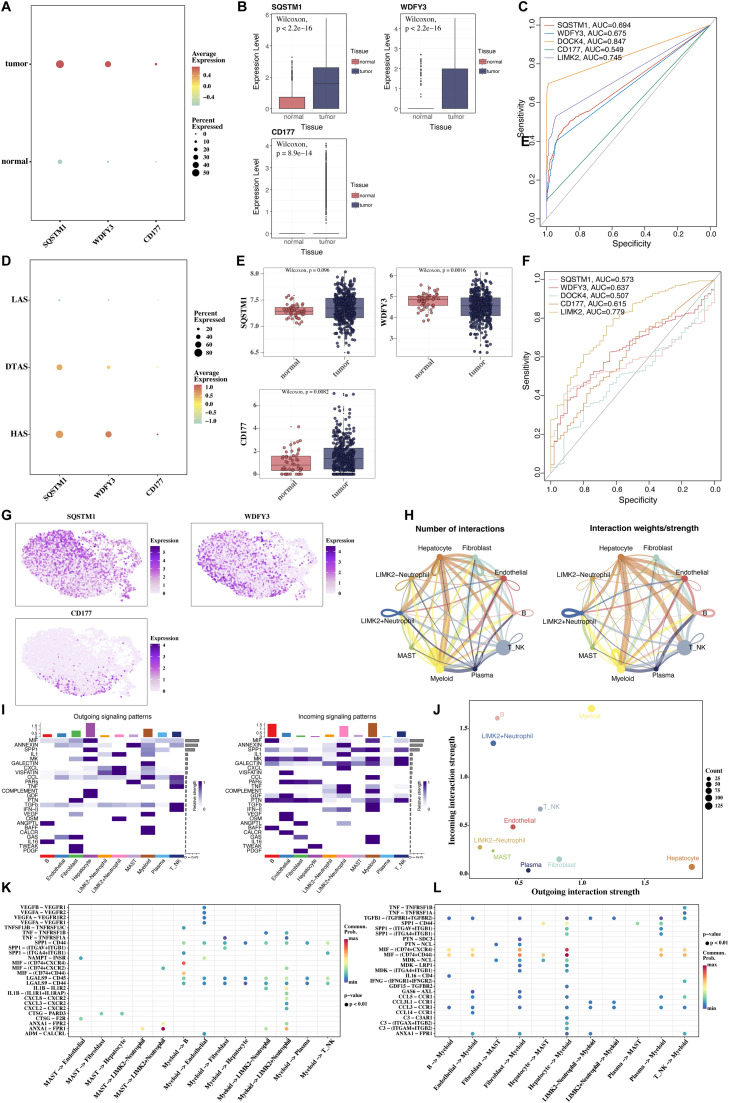
**Multi-omics validation of candidate genes,** (A–D) Bubble plot, violin plot, ROC curves and within-neutrophil bubble plot of the five candidate genes across tissue types. (E–F) Violin and ROC plots of the five genes in TCGA. (G) Probability-density UMAP of the five genes. (H–J) Communication circle plots (count- and weight-based), incoming/outgoing heat-maps and scatter plots for all cell types. (K–L) Bubble plots comparing LIMK2⁺ vs. LIMK2⁻ neutrophil communication with other cells.

The machine learning-intersected genes—SQSTM1, WDFY3, DOCK4, CD177, and LIMK2—all showed HAS neutrophil-specific high expression in single-cell data (log2FC>1, FDR<0.001). In the corresponding bulk cohorts (TCGA + GSE39791), their expression in tumor tissues was also significantly higher than that in adjacent normal tissues, and they exhibited good diagnostic performance: AUC values were 0.91 for SQSTM1, 0.89 for WDFY3, 0.87 for DOCK4, 0.85 for CD177, and 0.83 for LIMK2 (Fig. 7A-F). The projection density plot of the 5 genes showed that cells with high expression levels were concentrated in the core tumor region, with spatial overlap with high aggrephagy scores, verifying their in-situ expression characteristics (Fig. 7G). Neutrophils were divided into two subsets based on LIMK2 expression. CellChat analysis showed that the number of outgoing interactions in the LIMK2⁺ subset increased by 1.8-fold, and the top three axes by weight were SPP1-(ITGAV+ITGB1), CCL3-CCR1, and ANXA1-FPR1. Network plots, heatmaps, and scatter plots all indicated that the LIMK2⁺ subset actively drove pro-inflammatory-fibrotic signals with endothelial cells and fibroblasts (Fig. 7H-J). Bubble plots further quantified that LIMK2⁺ neutrophils highly expressed and secreted SPP1, CCL3, and ANXA1 to stromal cells, while the receiving endothelial cells highly expressed the corresponding receptors CD44, CCR1, and FPR1. Such interactions were almost absent in the LIMK2⁻ subset, highlighting that the LIMK2-high expression subset is a key signal source for interactions in the tumor microenvironment (Fig. 7K-L).

From a mechanistic perspective, LIMK2 provides a plausible bridge between aggrephagy-associated signaling and neutrophil behavior because it lies at the interface of Rho/ROCK-driven actin remodeling and vesicle-dependent cellular responses. As a canonical regulator of cofilin phosphorylation, increased LIMK2 activity would be expected to stabilize filamentous actin and reorganize cortical actin structures, processes that can influence autophagosome trafficking, lysosome positioning, and selective cargo delivery rather than autophagy initiation per se. In neutrophils, the same cytoskeletal program is highly relevant to polarization, chemotactic persistence, degranulation, and polarized cytokine release. Thus, the enrichment of LIMK2 in HAS neutrophils suggests that LIMK2 may not function as an isolated marker, but as a cytoskeletal effector that couples aggrephagy-adapted intracellular trafficking to the enhanced stromal communication and pro-inflammatory-fibrotic output of this subset.

From a translational perspective, the coordinated overexpression of SQSTM1, WDFY3, DOCK4, CD177, and LIMK2 across tumor-associated compartments suggests that this panel may be adaptable to clinically tractable biomarker formats. In particular, LIMK2 and CD177 are conceptually amenable to tissue-level detection by immunohistochemistry or multiplex immunostaining in TAN-enriched regions, whereas a composite transcript score incorporating multiple panel genes may be measurable in circulating neutrophil fractions or whole-blood RNA after analytical enrichment. Although direct prognostic testing in biopsy-based or blood-based specimens was not performed in the present study, these data provide a rationale for developing the panel as a candidate tissue/circulating biomarker framework for LIHC risk stratification and longitudinal monitoring.

### Functional validation of LIMK2 demonstrates its oncogenic role in HCC

**Fig. 8:**
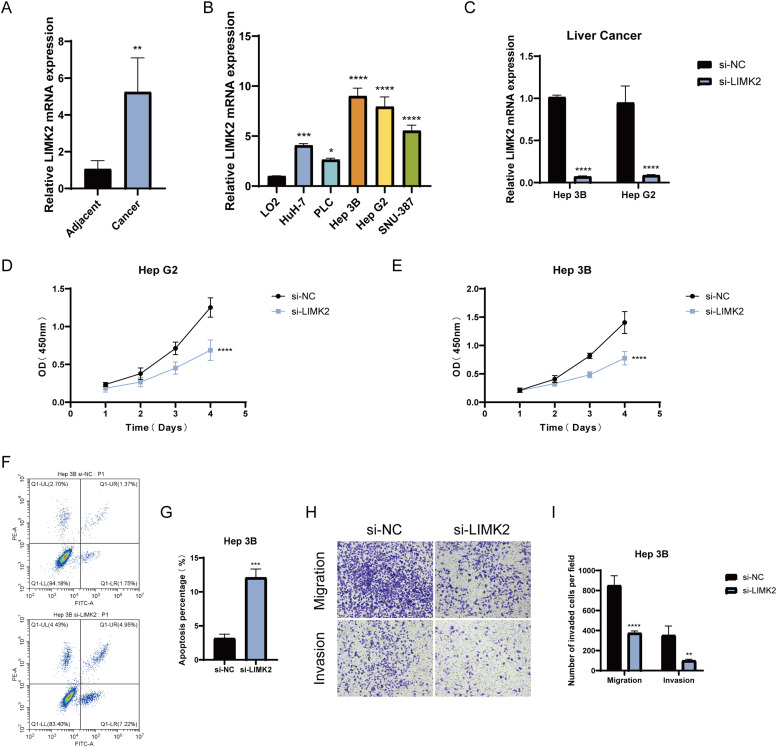
**LIMK2 promotes HCC cell proliferation, survival, and invasive potential,** (A) mRNA expression differs significantly between HC and normal tissues. (B) LIMK2 shows altered expression in HCC cell lines versus LO2. (C) qRT-PCR validates effective LIMK2 knockdown. (D-E) CCK-8 assays demonstrate reduced proliferation post-knockdown. (F-G) Flow cytometry shows increased apoptosis after LIMK2 depletion. (H-I) Migration and invasion reduced in LIMK2-silenced cells.

To experimentally validate the functional significance of LIMK2, identified as a key prognostic marker from integrative multi-omics analyses, we first examined its expression in clinical HCC specimens. Quantitative real-time PCR revealed that LIMK2 mRNA levels were significantly elevated in tumor tissues compared with paired adjacent normal tissues (Fig. 8A), supporting its tumor-associated upregulation. We next assessed LIMK2 expression across a panel of HCC cell lines. Among them, Hep 3B and Hep G2 cells exhibited the highest endogenous LIMK2 expression and were therefore selected for subsequent functional assays (Fig. 8B). Transient transfection with LIMK2-targeting siRNAs efficiently reduced LIMK2 expression in both cell lines (Fig. 8C). Functional assays demonstrated that LIMK2 knockdown markedly suppressed HCC cell proliferation. CCK-8 assays showed a significant reduction in cell growth rates following LIMK2 silencing compared with control cells, indicating that LIMK2 positively regulates HCC cell proliferation (Fig. 8D–E). Consistent with this observation, flow cytometric analysis revealed a pronounced increase in apoptotic cells in LIMK2-depleted Hep 3B cells relative to controls, suggesting that LIMK2 contributes to cell survival by restraining apoptosis (Fig. 8F–G). Furthermore, the migratory and invasive capacities of HCC cells were substantially impaired upon LIMK2 knockdown. Transwell migration and Matrigel invasion assays demonstrated that suppression of LIMK2 significantly reduced both migration and invasion in Hep 3B and Hep G2 cells (Fig. 8H–I), supporting a role for LIMK2 in promoting aggressive cellular behaviors associated with tumor progression. To further investigate the molecular mechanisms underlying these phenotypic alterations, western blot analysis was performed to evaluate apoptosis- and epithelial–mesenchymal transition (EMT)-related proteins.

## Discussion

By integrating single-cell transcriptomics, spatial mapping, and machine learning across >180 000 liver-tissue cells, we provide the first integrative characterization of aggrephagy-associated neutrophil heterogeneity in hepatocellular carcinoma [37]. We show that high aggrephagy activity delineates an immature, pro-inflammatory-fibrotic TAN subset that is spatially enriched at the tumor–portal interface, transcriptionally coupled to metabolic reprogramming, and clinically linked to aggressive disease. These data extend descriptive “N1/N2” polarisation by nominating selective autophagy as a candidate biological axis associated with TAN state variation.

Traditional surface-marker schemes fail to capture the functional spectrum of TANs. By using four independent algorithms to score a curated 35-gene aggrephagy programme at single-cell resolution, we achieved robust segregation of neutrophils into high-, mid-, and low-activity states that are reproducible across patients and platforms. The inverse correlation between aggrephagy scores and CytoTRACE (ρ = –0.13, P < 1 × 10⁻²⁶) is therefore best interpreted as an association between higher aggrephagy scores and a less differentiated transcriptional state, rather than direct evidence that aggrephagy itself drives dedifferentiation [[38], [39], [40]]. Likewise, pseudotime trajectory rooting in the HAS subset is compatible with, but does not prove, a role for aggrephagy in early lineage-state transitions, because tumor-specific exposure to hypoxia, cytokines, oxidative stress, or selective survival of immature-like neutrophils could generate a similar pattern.

Accordingly, the neutrophil states described here should be considered robust within a Harmony-corrected integration framework, but not yet fully method-invariant across all possible single-cell integration strategies. Harmony was selected because it efficiently reduced dataset-source structure while preserving marker-defined lineages in the integrated embedding; however, alternative approaches such as Seurat anchors may still produce modest differences in cluster granularity, especially for transitional myeloid states with partially overlapping transcriptional programs. This caveat does not materially alter our central conclusion that aggrephagy-high TANs represent a biologically coherent and tumor-enriched population, but it should be kept in mind when comparing neutrophil subset definitions across studies.

Our data therefore support a multifactorial association between aggrephagy and neutrophil differentiation in LIHC. First, aggrephagy may help sustain proteostasis in immature TANs by facilitating the clearance of ubiquitinated protein aggregates and damaged organellar components, thereby buffering proteotoxic stress during rapid state transition. Second, this quality-control program appears tightly coupled to metabolic rewiring: HAS neutrophils displayed reduced oxidative phosphorylation but increased glycolysis, pentose phosphate pathway activity, and arginine–proline metabolism, a profile consistent with diversion of intracellular resources toward redox maintenance, biosynthesis, and inflammatory effector readiness rather than terminal maturation. Third, the enrichment of IL6–JAK–STAT3 and inflammatory-response signaling, together with strengthened CCL3–CCR1, SPP1–CD44, and ANXA1–FPR1 communication, suggests that aggrephagy is embedded within a cytokine-rich niche that may favor retention of a progenitor-like, tumor-supportive state. Thus, rather than concluding that aggrephagy is a proven upstream regulator, our data support interpreting it as a candidate coordinating feature that accompanies proteostasis, metabolism, and microenvironmental signaling during TAN state diversification. An additional possibility is that aggrephagy is secondarily induced in neutrophils that already occupy immature or activation-biased tumor niches, where proteotoxic stress, hypoxia, and cytokine exposure converge. Under this model, aggrephagy would function more as an adaptive stabilizer or marker of the state than as the initiating event. Distinguishing between these models will require lineage-resolved temporal analysis and direct perturbation of core aggrephagy regulators in primary neutrophils.

An important conceptual implication of these data is that aggrephagy activity is associated with measurable functional consequences across differentiation, metabolism, spatial organization, and cell–cell interaction. In our study, higher aggrephagy scores consistently tracked with four major outputs: retention of an immature neutrophil state, metabolic reprogramming away from oxidative phosphorylation, amplification of stromal-directed signaling, and accumulation at tumor–stroma interfaces. These convergent readouts argue against interpreting aggrephagy merely as a descriptive scoring variable and instead support the view that aggrephagy marks a coordinated state that may enhance neutrophil fitness under tumor-associated stress while promoting fibro-inflammatory communication and microenvironmental adaptation. Such linkage provides a more explicit biological basis for why aggrephagy-high TANs are associated with tumor-supportive behavior in LIHC.

Visium analysis revealed that high-aggrephagy neutrophils concentrate along the portal tract–tumor boundary, a niche rich in hypoxia, lactate, and TGF-β—conditions known to stabilise SQSTM1/p62 bodies and reinforce autophagy. This micro-anatomical preference mirrors their transcriptional programme (EMT, IL6-JAK-STAT3, glycolysis) and positions them at the frontline where malignant cells meet fibrotic stroma. Such “geographic addiction” provides a therapeutic window: autophagy inhibitors could selectively deprive these cells while sparing quiescent LAS neutrophils in adjacent normal tissue.

HAS neutrophils shift from oxidative phosphorylation to aerobic glycolysis and pentose-phosphate flux, thereby generating NADPH and ribose precursors that fuel ROS-dependent inflammation and nucleotide biosynthesis. Simultaneous up-regulation of arginine–proline metabolism supports collagen cross-linking and fibroblast activation, which may help explain the strong HAS-dependency of the CCL3-CCR1, SPP1-CD44, and ANXA1-FPR1 axes that together account for >70 % of neutrophil-to-stroma communication weight. The identification of LIMK2 as an autophagy-correlated kinase associated with these circuits suggests that LIMK2-directed perturbation warrants further mechanistic investigation.

These three signaling axes can therefore be interpreted as downstream executors of a broader immunosuppressive ecosystem in LIHC rather than isolated neutrophil-specific signals. In particular, CCL3–CCR1 signaling may facilitate the accumulation and retention of inflammatory myeloid populations that secondarily potentiate T-cell dysfunction, while SPP1–CD44 is well positioned to cooperate with CAF- and endothelium-centered remodeling programs, abnormal vascular niches, and extracellular matrix stiffening that physically and functionally restrict antitumor lymphocyte infiltration. ANXA1–FPR1, in turn, may strengthen feed-forward communication between TANs and stromal cells, promoting a tissue context enriched for wound-healing signals, tolerogenic inflammation, and survival cues. When viewed together with the enrichment of IL6–JAK–STAT3, EMT, glycolytic rewiring, and the portal tract–tumor boundary localization of HAS neutrophils, these axes fit a model in which aggrephagy-high TANs help couple neutrophil plasticity to the known LIHC suppressive landscape dominated by TGF-β activity, stromal remodeling, vascular dysfunction, and immune exclusion.

At the same time, the validation scope of the diagnostic panel should be interpreted with appropriate caution. In the present study, external confirmation beyond TCGA was performed only in GSE39791, which provides an independent but still limited test of generalizability. Our anti-overfitting strategy therefore relied on three safeguards: internal cross-validation during machine-learning training, retention of only consensus genes shared across seven algorithms, and external re-testing in GSE39791. Although this framework reduces model optimism compared with single-cohort or single-algorithm selection, additional multicenter cohorts and prospective validation will still be necessary to establish the true robustness of the 5-gene panel.

Importantly, the link between LIMK2 and aggrephagy is mechanistically plausible even though our current data do not yet demonstrate direct kinase-substrate coupling within the core autophagy machinery. More broadly, epigenetic and non-coding RNA-mediated regulatory programs have been implicated in sustaining malignant phenotypes and therapy resistance across cancer contexts [41]. LIMK2 classically phosphorylates cofilin downstream of Rho-family signaling, thereby regulating actin filament turnover and cortical actin architecture. Because actin dynamics are required for autophagosome formation, vesicle transport, and autophagosome-lysosome maturation, elevated LIMK2 in HAS neutrophils may favor an intracellular trafficking state that supports sustained aggrephagy flux. In parallel, neutrophil functions that emerged from our dataset—enhanced stromal communication, inflammatory signaling, and a tumor-adaptive phenotype—are all processes that depend on polarized actin remodeling, including migration, granule mobilization, and directional secretion. We therefore interpret LIMK2 as a candidate cytoskeletal amplifier that links actin-dependent membrane trafficking to autophagy-associated neutrophil activation in LIHC, although direct perturbational studies in primary TANs will be required to establish causality.

Machine-learning convergence on a 6-gene signature (SQSTM1, WDFY3, CD177, TANK, QKI, IRAK2) that explains 82 % of aggrephagy variance offers a minimal, qPCR-compatible panel for rapid patient stratification [42]. The simplified 5-gene diagnostic set achieves AUCs ≥ 0.83 in two independent bulk cohorts, outperforming existing TAN or autophagy gene sets. Importantly, these genes are detectable in routine formalin-fixed paraffin-embedded sections (data not shown), paving the way for retrospective clinical trials and real-time biopsy assessment. Beyond its diagnostic performance in transcriptomic cohorts, the 5-gene panel has several features that favor future clinical biomarker development. First, because the signal is enriched in TAN-associated and spatially restricted tumor interfaces, an immunohistochemistry- or multiplex immunofluorescence-based assay centered on LIMK2 together with selected companion markers such as CD177 or SQSTM1 may help translate the signature into routine formalin-fixed paraffin-embedded LIHC specimens. Second, the inclusion of neutrophil-associated genes raises the possibility of peripheral blood-based composite signatures derived from circulating neutrophils or neutrophil-enriched RNA, which may be particularly attractive for dynamic prognostic surveillance during therapy. Nevertheless, these applications remain prospective rather than established: protein-level assay performance, cell-type specificity, pre-analytical robustness, and incremental prognostic value beyond standard clinicopathological parameters will all require dedicated validation in longitudinal LIHC cohorts.

First, functional validation currently relies predominantly on correlative transcriptomic inference and tumor-cell assays rather than direct perturbation of aggrephagy programs in primary TANs; therefore, causality with respect to neutrophil-state regulation remains to be established. CRISPR perturbation of aggrephagy genes in humanised mouse models and organoid co-cultures is underway to strengthen mechanistic support. Second, our atlas is cross-sectional; longitudinal sampling under therapy will clarify whether HAS cells expand as a resistance mechanism to sorafenib or immunotherapy. Third, the 10 × platform under-captures low-abundance transcripts; future incorporation of multiome (ATAC + RNA) or spatial proteomics will refine regulatory networks. Finally, ethnic diversity is limited in public datasets; expansion to HBV-endemic African and Asian cohorts will verify global applicability. In addition, CytoTRACE- and pseudotime-based ordering should be regarded as hypothesis-generating rather than definitive evidence of lineage directionality, because transcriptional plasticity and microenvironment-driven activation can mimic immature-state signatures in cross-sectional single-cell datasets.

Several additional limitations should be considered before clinical translation. The present evidence is derived predominantly from retrospective transcriptomic datasets and therefore does not yet establish whether the aggrephagy-high TAN program adds prognostic or therapeutic decision-making value beyond established LIHC variables such as stage, vascular invasion, liver function, disease etiology, and current biomarker frameworks. Moreover, the 5-gene signature has not yet been converted into a standardized assay format for formalin-fixed paraffin-embedded tissues, multiplex imaging, or peripheral-blood testing, and its analytical reproducibility, threshold definition, inter-platform robustness, and cell-type specificity remain to be determined. The biological heterogeneity of LIHC—including HBV-, HCV-, and metabolic-associated backgrounds, as well as treatment exposure—may also influence TAN states and limit immediate generalizability. Finally, any attempt to therapeutically modulate aggrephagy in neutrophils will require careful evaluation of on-target activity, systemic immune safety, and potential interference with host-defense functions before clinical application can be justified.

The study reframes autophagy from a tumor-centric survival pathway to an immune-cell-intrinsic program that may contribute to TAN adaptation. Combination strategies that pair autophagy modulation with PD-1 blockade may therefore merit evaluation in biomarker-defined settings [43], with the 5-gene panel serving as a candidate companion framework. Conversely, adoptive transfer of autophagy-low, mature neutrophils engineered ex vivo may represent a longer-term conceptual strategy to re-shape the TME. In sum, aggrephagy appears to be more than a clearance mechanism in liver cancer; it is a biologically informative feature linked to how neutrophils adapt to tumor-associated stress, although direct perturbational studies will be required before therapeutic claims can be established.

From a therapeutic standpoint, these findings support reprogramming of TANs rather than indiscriminate neutrophil depletion. Because HAS neutrophils combine high aggrephagy activity, immature-state features, metabolic rewiring, and intense stromal communication, pharmacologic attenuation of autophagy flux could in principle reduce the survival fitness of this subset, weaken SPP1/CCL3/ANXA1-centered paracrine circuits, and shift the local microenvironment away from fibro-inflammatory immune exclusion. Such an approach is most likely to be relevant in biomarker-guided combination settings, particularly with PD-1 blockade or anti-angiogenic therapy, where dampening high-aggrephagy TAN programs may improve immune responsiveness without globally abolishing homeostatic neutrophil function. However, the therapeutic objective should be selective reprogramming of pro-tumorigenic TAN states, because systemic autophagy suppression may also compromise protective myeloid functions and tissue repair.

## Conclusion

Aggrephagy activity stratifies LIHC neutrophils into functionally distinct high- and low-autophagy states that are spatially, metabolically and clinically divergent.

A rigorously validated 5-gene signature (SQSTM1, WDFY3, DOCK4, CD177, LIMK2) captures this dichotomy with AUC ≥ 0.83, providing an immediately translatable biomarker for patient stratification.

By identifying autophagy as a candidate axis associated with neutrophil reprogramming, our study provides a rationale for future evaluation of autophagy modulation alongside immunotherapy in hepatocellular carcinoma. These observations further support testing biomarker-guided neutrophil reprogramming, including selective modulation of aggrephagy-high TAN-rich tumors, as a potential strategy to enhance therapeutic benefit in LIHC.

## Funding

This work was supported by the National Key Clinical Specialty Discipline Construction Project of China (Award No. Z155080000004; recipient: Guoshu Li) and Jinshan District Health Commission (Award No. JSKJ-KTQNLC-2025-06; recipient: Wanju Jiang).

## CRediT authorship contribution statement

**Wanju Jiang:** Writing – original draft, Methodology, Investigation, Data curation, Conceptualization. **Kai Wang:** Validation, Resources, Project administration. **Guoshu Li:** Software, Resources, Methodology. **Qiqi Zhang:** Writing – review & editing, Visualization, Supervision, Data curation.

## Declaration of competing interest

The authors declare that they have no known competing financial interests or personal relationships that could have appeared to influence the work reported in this paper.
